# Financial hardship after COVID-19 infection among US Veterans: a national prospective cohort study

**DOI:** 10.1186/s12913-024-11421-1

**Published:** 2024-08-19

**Authors:** Diana J. Govier, David P. Bui, Katrina E. Hauschildt, Tammy L. Eaton, Holly McCready, Valerie A. Smith, Thomas F. Osborne, C. Barrett Bowling, Edward J. Boyko, George N. Ioannou, Matthew L. Maciejewski, Ann M. O’Hare, Elizabeth M. Viglianti, Amy S. B. Bohnert, Denise M. Hynes, Theodore J. Iwashyna

**Affiliations:** 1 Center to Improve Veteran Involvement in Care (CIVIC), VA Portland Health Care System, 3710 SW US Veterans Hospital Rd., Portland, OR 97239 USA; 2 Oregon Health & Science University – Portland State University School of Public Health, Portland, OR USA; 3 VA Center for Clinical Management Research, VA Ann Arbor Health Care System, Ann Arbor, MI USA; 4 School of Medicine, Johns Hopkins University, Baltimore, MD USA; 5 University of Michigan Institute for Healthcare Policy & Innovation, Ann Arbor, MI USA; 6 Center of Innovation to Accelerate Discovery and Practice Transformation, VA Durham Health Care System, Durham, NC USA; 7 Department of Medicine, Duke University, Durham, NC USA; 8 Department of Population Health Sciences, Duke University School of Medicine, Durham, NC USA; 9 VA Palo Alto Health Care System, Palo Alto, CA USA; 10 Department of Radiology, Stanford University School of Medicine, Stanford, CA USA; 11 Durham Veterans Affairs Geriatric Research Education and Clinical Center, VA Durham Health Care System, Durham, NC USA; 12 Seattle Epidemiologic Research and Information Center, VA Puget Sound Health Care System, Seattle, WA USA; 13 Center of Innovation for Veteran-Centered and Value-Driven Care, VA Puget Sound Health Care System, Seattle, WA USA; 14 Division of Gastroenterology, Department of Medicine, University of Washington, Seattle, WA USA; 15 Hospital and Specialty Medicine Service, VA Puget Sound Health Care System, Seattle, WA USA; 16 Division of Nephrology, Department of Medicine, University of Washington, Seattle, WA USA; 17 Division of Pulmonary and Critical Care Medicine, Department of Internal Medicine, University of Michigan, Ann Arbor, MI USA; 18 Department of Psychiatry, University of Michigan Medical School, Ann Arbor, MI USA; 19 College of Health and Center for Quantitative Life Sciences, Oregon State University, Corvallis, OR USA; 20 School of Nursing, Oregon Health & Science University, Portland, OR USA; 21 Department of Medicine, University of Michigan Medical School, Ann Arbor, MI USA; 22 School of Public Health, Johns Hopkins University, Baltimore, MD USA

**Keywords:** COVID-19, SARS-CoV-2, Financial hardship, Veterans, Cohort study, Survey, Emulated target trial

## Abstract

**Background:**

Research suggests an association between COVID-19 infection and certain financial hardships in the shorter term and among single-state and privately insured samples. Whether COVID-19 is associated with financial hardship in the longer-term or among socially vulnerable populations is unknown. Therefore, we examined whether COVID-19 was associated with a range of financial hardships 18 months after initial infection among a national cohort of Veterans enrolled in the Veterans Health Administration (VHA)—the largest national integrated health system in the US. We additionally explored the association between Veteran characteristics and financial hardship during the pandemic, irrespective of COVID-19.

**Methods:**

We conducted a prospective, telephone-based survey. Out of 600 Veterans with COVID-19 from October 2020 through April 2021 who were invited to participate, 194 Veterans with COVID-19 and 194 matched comparators without a history of infection participated. Financial hardship outcomes included overall health-related financial strain, two behavioral financial hardships (e.g., taking less medication than prescribed due to cost), and seven material financial hardships (e.g., using up most or all savings). Weighted generalized estimating equations were used to estimate risk ratios (RR) and 95% confidence intervals (CI) of financial hardship by COVID-19 status, and to assess the relationship between infection and Veteran age, VHA copay status, and comorbidity score, irrespective of COVID-19 status.

**Results:**

Among 388 respondents, 67% reported at least one type of financial hardship since March 2020, with 21% reporting behavioral hardships and 64% material hardships; 8% reported severe-to-extreme health-related financial strain. Compared with uninfected matched comparators, Veterans with a history of COVID-19 had greater risks of severe-to-extreme health-related financial strain (RR: 4.0, CI: 1.4–11.2), taking less medication due to cost (RR: 2.9, 95% CI: 1.0–8.6), and having a loved one take time off work to care for them (RR: 1.9, CI: 1.1–3.6). Irrespective of COVID-19 status, Veterans aged < 65 years had a greater risk of most financial hardships compared with Veterans aged ≥ 65 years.

**Conclusions:**

Health-related financial hardships such as taking less medication due to cost and severe-to-extreme health-related financial strain were more common among Veterans with a history of COVID-19 than among matched comparators. Strategies are needed to address health-related financial hardship after COVID-19.

**Trial registration:**

NCT05394025, registered 05–27-2022.

**Supplementary Information:**

The online version contains supplementary material available at 10.1186/s12913-024-11421-1.

## Background

The economic ramifications of the COVID-19 pandemic have been felt across the United States (US) [1]. Indeed, during the pandemic’s first year, approximately half of US adults lived in households where someone lost employment income and one-third had difficulty paying for usual expenses [2, 3]. Financial hardships including job loss, depletion of savings, and cost-related forgone medical care have also been documented among selected groups of patients within the first six months after SARS-CoV-2 infection (herein “COVID-19”) [4–7]. Across numerous health states and shocks, financial hardship is associated with declines in quality of life and health [8–10],  and later use of medical care [11]. Financial hardship has also been implicated in impaired recovery from severe COVID-19 [12].

Reflecting long-standing inequities, financial hardship during the COVID-19 pandemic has disproportionately affected older adults [13],  individuals with lower incomes [14], racial/ethnic minorities [3, 15], those living in the US south where the safety net is more limited (e.g., lack of Medicaid expansion, restrictive eligibility for social programs) [16], and those with severe COVID-19 [6, 7]. US Veterans who rely on the Veterans Health Administration (VHA) for their health care needs share many characteristics that place them at risk for financial hardship including older age, greater morbidity and disability burden, and higher prevalence of residing in the US South [17–21]. At the same time, younger Veterans tend to have higher incomes than their similar-aged non-Veteran peers, and many Veterans have VHA health care benefits, both potentially important sources of protection from financial hardship [22, 23].

To date, no studies have explored the association between COVID-19 infection and financial hardship among Veterans—a policy-relevant population in their own right, but also a population which provides information about financial hardship among a socially and medically vulnerable group. Further, prior studies on the relationship between COVID-19 and financial hardship have been limited by either short follow-up (i.e., ≤ 6 months); assessment of a relatively narrow set of financial hardship outcomes; study samples which were drawn from single states; privately-insured study samples whose experiences may not generalize to socially vulnerable populations; or lack of a comparison group without COVID-19 [4–7, 13], all of which is crucial information for identifying and addressing infection-related financial hardship. To begin to fill these knowledge gaps, we examined whether and the extent to which COVID-19 infection was associated with a range of behavioral and material financial hardships. We additionally explored the association between Veteran characteristics and financial hardship during the COVID-19 pandemic, irrespective of COVID-19 infection status.

## Methods

We conducted a prospective, telephone-based survey of VHA-enrolled Veterans with a documented history of COVID-19 infection and matched uninfected comparators. We focused on Veterans with infections that occurred between October 1, 2020 and April 30, 2021 (vs. very early or later in the pandemic) to allow for long-term follow-up and because this was a period when health care facility-based testing was common and self-testing was less frequent [24, 25], reducing the potential for misclassification of infection status. Secondary data from VHA electronic health records were used to match Veterans with COVID-19 to uninfected comparators. The survey was reviewed and approved by the VA Institutional Review Boards (IRBs) and Research & Development Committees of Ann Arbor and Durham VA medical centers; analyses of secondary data were reviewed and approved by the VA IRBs and Research & Development Committees of Ann Arbor, Durham, West Haven, Palo Alto, Portland, and Puget Sound VA medical centers. All participants provided verbal informed consent to participate in the survey. We followed STROBE reporting guidelines in reporting methods and results.

### Study population

The VA COVID-19 Observational Research Collaboratory (CORC)—a coordinated VA effort to support rigorous research on the long-term effects of COVID-19—utilized an emulated target trial framework [26] (see eTable 1, Additional File 1 for a comparison of the unethical target trial study design and emulated trial) to establish cohorts of VHA enrollees with documented COVID-19 infection and matched uninfected comparators using data from the VHA Corporate Data Warehouse (CDW) and COVID-19 Shared Data Resource (CSDR), which is a curated database focused on COVID-19 [27, 28]. To account for potential confounding between infection and long term outcomes, exact matching followed by propensity score matching was used based on 37 baseline sociodemographic, health and clinical, and health care utilization characteristics, which were selected by a multidisciplinary team of more than 30 subject matter experts including physicians, biostatisticians, and other COVID-19 scientists and using causal diagrams (see eTable 1, "Approach to balancing confounders", Additional File 1 for matching variables) [29]. Matching was done on a monthly basis from March 1, 2020 through April 30, 2021*.* To ensure a sufficiently deep pool of comparators from which to sample, we used a 25:1 matched design with replacement; Veterans without infection in a given month could be matched to more than one infected Veteran and were eligible for matching in subsequent months until infected.

### Survey methods

Approximately 18 months after initial infection, a random sample of 600 Veterans with COVID-19 infection—100 for each month of October through December 2020, and February through April 2021—were invited via mail to participate in a telephone-based survey to ascertain outcomes that are not traditionally measured in extant data, including financial hardship measures. To ensure broad representation, sampling was stratified by four US Census regions and probable or confirmed hospitalization for COVID-19. Among infected Veterans who completed surveys, we invited up to five of their best-matched comparators (matches 6–25 were not contacted) to participate in the survey until one was enrolled. To maximize response rates, we consented and interviewed respondents by telephone and offered to complete surveys over multiple sessions. All respondents, regardless of survey completion, were compensated $10. To minimize response and recall bias, surveys were administered by trained interviewers. Although interviewers were aware of Veterans’ COVID-19 status, the same survey was administered to Veterans with and without a history of COVID-19. Surveys were completed between May 23, 2022, and January 3, 2023.

Financial hardship measures were developed based on a World Health Organization (WHO) assessment instrument of cumulative health-related financial strain [30], and our previous work operationalizing financial hardship among Veterans and after critical illness [4, 6, 8, 16, 23]. To assess cumulative health-related financial strain, we asked respondents, since the beginning of the 2020 pandemic how much had their health been a drain on their financial resources, with response options including none, mild, moderate, severe, and extreme. From this, we defined health-related financial strain as reporting severe or extreme strain. We asked respondents two binary (yes, no) questions about their behavioral financial hardship experiences since the beginning of the 2020 pandemic, including whether they had skipped or delayed necessary medical care due to cost, or whether they had taken less medication than prescribed due to cost. We asked respondents seven binary (yes, no) questions about their material financial hardship since the beginning of the 2020 pandemic, including whether they had used up most or all of their savings, were contacted by a collections agency, had declared bankruptcy, were unable to pay for necessities, had lost a job, had to change the kind of work they could do, or whether a loved one had to take time off to care for them. From these, we created two binary global outcomes for any behavioral financial hardship and any material financial hardship. Lastly, we categorized respondents as having any financial hardship if they responded in the affirmative to any of the nine close-ended behavioral or material financial hardship questions or reported severe-to-extreme health-related financial strain. See eTable 2, Additional File 1 for a list of survey questions and response options.

In addition to survey data, we used demographic (e.g., age, sex, race), clinical (e.g., comorbidity scores), and healthcare use data (e.g., outpatient visits) from respondents’ electronic healthcare records to describe and compare our study participants.

### Statistical methods

We created analytic weights for sampling and survey nonresponse to weight our sample to represent the full CORC cohort. We used multivariable logistic regression to estimate the probability of nonresponse among eligible participants with COVID-19 who were initially invited to participate. The model for nonresponse included age, sex, race, comorbidity score via Gagne index [31], and other variables selected from a lasso procedure which considered all variables used in cohort matching. The final analytic weight used was the inverse product of the probability of sampling and responding to the survey. All analyses and results incorporate these analytic weights, and we used weighted descriptive statistics to report sample characteristics. 

We calculated weighted prevalence estimates of financial hardship with logit-transformed confidence intervals [32]. In addition, we estimated risk ratios to compare the risk of prevalent financial hardship between Veterans with a history of COVID-19 and uninfected comparators, as well as by selected sociodemographic and clinical factors―baseline age group (< 65 years, ≥ 65 years), morbidity score (Nosos risk adjustment score ≤ 1 and > 1), and VHA copay status (none, some/full). Nosos score is a measure of expected annual VA health care costs for an individual, with values > 1 representing above average expected health care costs [33]. Veterans were categorized into copay groups using data on copays for VHA medical care (the majority of Veterans have copays for medications) and VHA priority benefit groups [34, 35]. Models were fitted with weighted generalized estimating equations (GEEs), with a Poisson family and log link. In addition, models included an exchangeable correlation structure and 95% confidence intervals (CIs) with robust standard errors clustered on match group. Each model included a single binary independent variable for the modeled factor [e.g., COVID-19 status (yes/no)]; we did not adjust for additional covariates as we found generally good balance in baseline characteristics (SMDs < 0.20) between groups.

With one exception, all analyses utilized the full analytic sample. For the Nosos risk-adjustment score analysis, 10 individuals with missing Nosos scores were excluded. All analyses were conducted in R (version 4.3.1).

We tested the sensitivity of our results to cross-over infection among comparators by excluding eight matched pairs (16 participants) in which the comparator without a history of COVID-19 had a documented infection sometime during the follow-up period and before survey administration. We observed no differences in the sign or significance of our results when excluding these matched pairs (see eTables 7 and 8 in Additional File 1).

## Results

**Fig. 1:**
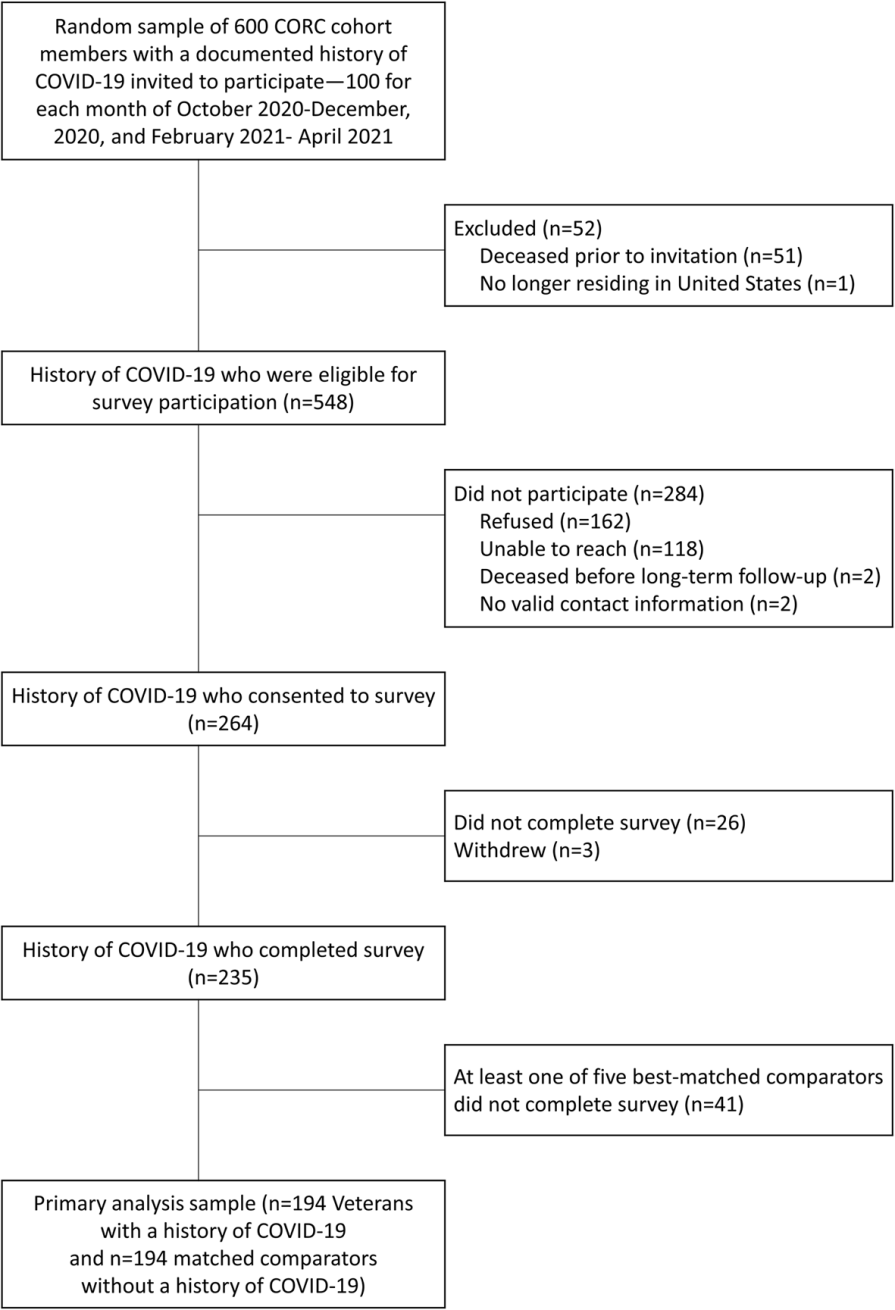
Study Flow Diagram

Of 231,160 Veterans with COVID-19 in the original cohort, 600 were identified in our stratified random sample (Fig. 1). Among them, 548 were alive and living in the U.S. at the time of survey fielding; 194 (35%) completed surveys along with 194 matched comparators, yielding an analytic sample of 388. Differences between survey respondents and non-respondents were small. However, respondents tended to have more primary care contacts in the prior two years than non-respondents (mean = 10.2 and 8.3, respectively, data not shown).

### Sample characteristics

Our sample was primarily male (91%) and had an average age of 60.9 years (SD 13.8) (after weighting, Table 1), which is characteristic of the VHA population [36]. Most resided in urban areas (68%)*.* Over half had no VHA copays (63%) and most had Nosos risk adjustment scores ≤ 1 (54%). Veterans were well-connected with VHA, with a mean of 10.7 (SD 11.1) VHA primary care contacts during the two years prior. Those with and without a history of COVID-19 were well-balanced on most observed characteristics, with small differences in baseline smoking status, urban residence, and mental health care utilization (SMDs 0.20—0.33).

**Table 1:** Weighted sample characteristics, overall and by COVID-19 status

**Baseline characteristic (weighted %)**	**Overall** ***N* = 388**	**History of COVID-19** ***N *= 194**	**Matched comparator** ***N*** **= 194**	**SMD**
**Used in matching**				
COVID-19	50.0	100.0	0.0	< 0.001
Sex				< 0.001
Female	9.1	9.1	9.1	
Male	90.8	90.8	90.8	
Unknown	0.1	0.1	0.1	
Age, years, mean (SD)	60.9 (13.8)	60.4 (13.7)	61.4 (14.0)	-0.074
Race				0.129
Black or African American	26.2	29.0	23.3	
White	68.1	65.5	70.6	
Other	5.8	5.5	6.1	
Ethnicity				0.172
Hispanic	6.6	4.7	8.5	
Not Hispanic	91.4	92.7	90.1	
Unknown	2.0	2.6	1.4	
Smoking status				0.203
Current	10.6	9.4	11.7	
Former	44.7	47.3	42.1	
Never	39.6	40.1	39.1	
Unknown	5.1	3.2	7.1	
Vaccinated for COVID-19 January—April 2021				< 0.001
Unvaccinated	26.8	26.8	26.8	
Vaccinated	2.1	2.1	2.1	
Vaccine unavailable at index date	71.1	71.1	71.1	
Urban residence	67.5	72.7	62.3	0.223
Nosos risk-adjustment score				0.065
≤ 1	53.5	54.1	52.9	
> 1	45.7	45.4	46.0	
Unknown	0.8	0.5	1.0	
Immunosuppressed	10.6	10.6	10.6	< 0.001
No. VHA inpatient admissions, mean (SD)	0.4 (1.1)	0.3 (1.0)	0.4 (1.2)	-0.091
No. VHA primary care visits, mean (SD)	10.7 (11.1)	10.6 (10.6)	10.9 (11.6)	-0.023
No. VHA specialty care visits, mean (SD)	16.6 (17.1)	16.1 (15.3)	17.2 (18.8)	-0.068
No. VHA mental health visits, mean (SD)	6.3 (16.4)	8.9 (20.9)	3.6 (9.4)	0.326
BMI, mean (SD)	31.7 (6.6)	31.0 (5.6)	32.4 (7.5)	-0.225
Gagne index, mean (SD)	1.2 (2.0)	1.3 (1.8)	1.1 (2.2)	0.073
CAN score, mean (SD)	57.0 (28.1)	58.0 (28.8)	55.9 (27.5)	0.075
**Not used in matching**				
No. CDC high-risk conditions, mean (SD)	2.1 (1.7)	2.1 (1.6)	2.1 (1.8)	-0.003
No. CDC high-risk mental health conditions, mean (SD)	0.8 (1.0)	0.8 (1.0)	0.8 (1.0)	0.054
Distance to nearest VAMC, miles, mean (SD)	35.3 (35.4)	33.3 (36.9)	37.3 (33.8)	-0.114
Hospitalized at index date (± 7 days)	5.6	9.8	2.0	0.336
VHA copay group				0.147
Full copay	17.7	16.2	19.1	
Some copay	19.4	22.2	16.7	
No copay	62.9	61.6	64.3	
Area Deprivation Index, mean (SD)	56.1 (24.4)	56.5 (26.2)	55.8 (22.5)	0.027
Area Deprivation Index Median				0.046
Lower Median (1–50)	44.4	45.6	43.2	
Upper Median (51–100)	55.6	54.4	56.8	
**Characteristic at time of survey completion (weighted %)**	**Overall** ***N* = 388**	**History of COVID-19** ***N*** **= 194**	**Matched comparator** ***N*** **= 194**	**SMD**
Employment status				0.460
Currently working	37.2	39.4	35.1	
Unemployed and looking for work	5.5	2.6	8.3	
Disabled	16.5	23.1	10.0	
Retired/Other	40.8	34.9	46.6	

Other baseline variables used in matching but not reported to preserve table brevity include index month, state of residence, and indicators for nursing home residence, coronary heart disease, cancer (excluding non-metastatic skin cancers), chronic kidney disease, congestive heart failure, pulmonary-associated conditions (including asthma, COPD, interstitial lung disease, and cystic fibrosis), dementia, diabetes, hypertension, liver disease, sickle cell/thalassemia, solid organ or blood stem cell transplant, stroke/cerebrovascular disorders, substance use disorder, anxiety disorder, bipolar disorder, major depression, PTSD, and schizophrenia. Abbreviations: *SMD* Standardized mean difference, *BMI* Body Mass Index, *CAN* Care Assessment Need, *CDC* Centers for Disease Control and Prevention, *VHA* Veterans Health Administration, *VAMC* VA Medical Center, *SD* Standard Deviation

### Financial hardship

**Fig. 2:**
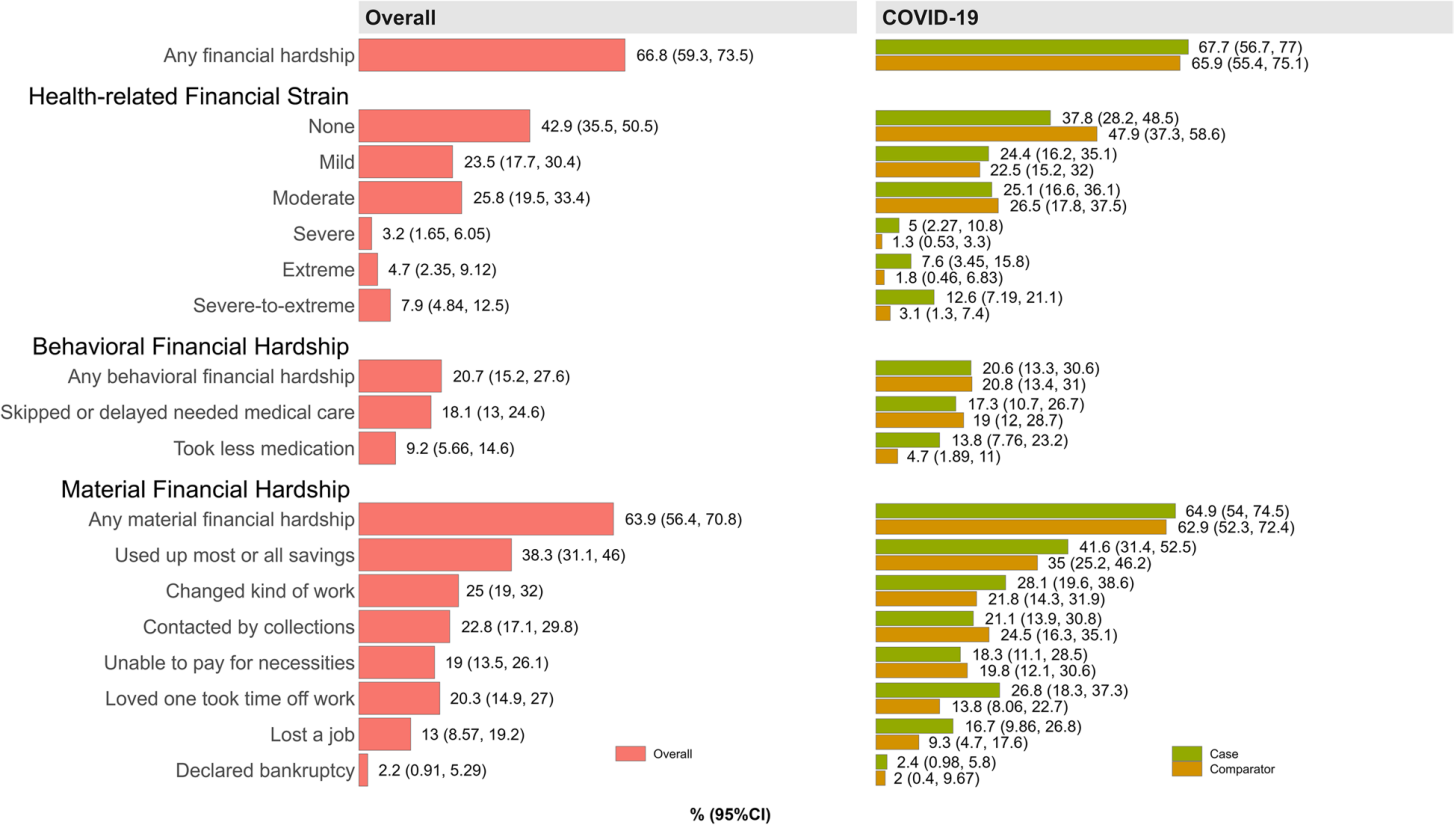
Financial hardship (Weighted Prevalence, 95% CI), overall and by COVID-19 status

Overall, 67% (95% CI: 59.3–73.5) of our sample reported experiencing any financial hardship during the pandemic, with 8% (95% CI: 4.8–12.5) reporting severe-to-extreme health-related financial strain (Fig. 2). Regarding behavioral hardship, skipping or delaying medical care due to cost was more common (18%, 95% CI: 13.0–24.6) than taking less medication due to cost (9%, 95% CI: 5.7–14.6). For material hardship, Veterans most frequently reported using up all or most savings (38%, 95% CI: 31.1–46.0) and having to change the kind of work they could do (25%, 95% CI: 19.0–32.0).

### COVID-19 infection and financial hardship

**Fig. 3:**
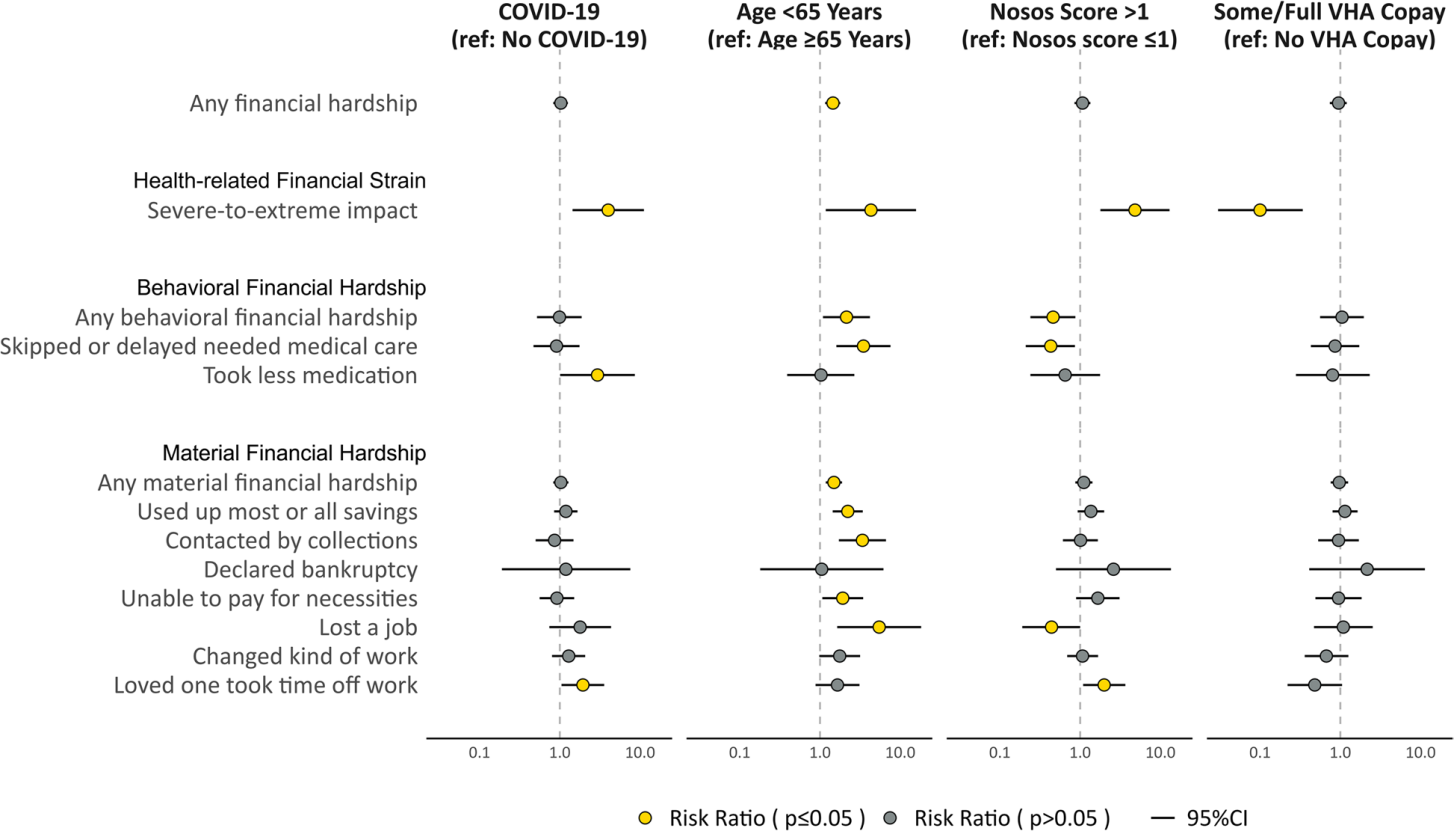
Financial hardship (Risk Ratio, 95% CI), by COVID-19 status and selected risk factors

Veterans with and without COVID-19 had similar likelihoods of experiencing any financial hardship [68% vs. 66%, respectively, relative risk (RR): 1.03, 95% CI: 0.8–1.3] and any behavioral financial hardship (21% among both groups, RR: 1.0, 95% CI: 0.5–1.9) (Figs. 2 and 3). However, Veterans with a history of COVID-19 more frequently reported taking less medication due to cost (14% vs. 5% of comparators, RR: 2.9, 95% CI: 1.0–8.6) and severe-to-extreme health-related financial strain (13% vs. 3% of comparators, RR: 4.0, 95% CI: 1.4–11.2). In addition, Veterans with and without COVID-19 had similar likelihoods of experiencing any material hardship (65% vs. 63%, respectively, RR: 1.0, 95% CI: 0.8–1.3). However, Veterans with a history of COVID-19 were more likely to report a loved one taking time off work to care for them (27% vs. 14% of comparators, RR: 1.9,  95% CI: 1.1–3.6) (see eTable 3, Additional File 1 for risk ratios and 95% CIs).

### Exploration of selected risk factors and financial hardship

**Fig. 4:**
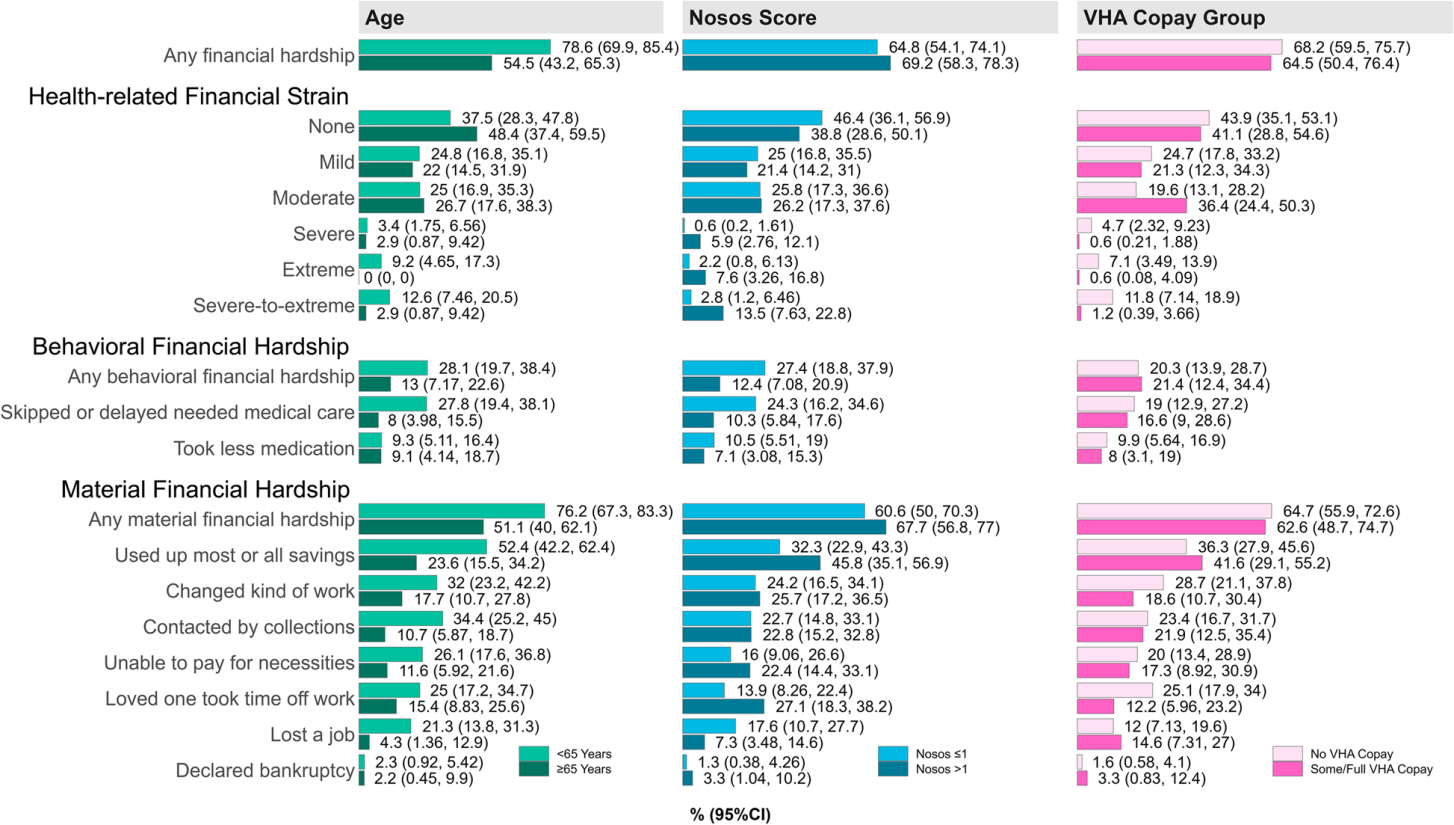
Financial hardship (Weighted Prevalence, 95% CI), by selected risk factors

Veterans aged < 65 years had a greater likelihood of experiencing any financial hardship than Veterans aged ≥ 65 years (79% vs. 55%, respectively, RR: 1.4, 95% CI: 1.2–1.8), any behavioral financial hardship (28% vs. 13%, RR: 2.1, 95% CI: 1.1–4.2), and skipping or delaying medical care due to cost since the beginning of the 2020 pandemic (28% vs. 8%, RR: 3.5, 95% CI: 1.6–7.6) (Figs. 3 and 4). Likewise, Veterans aged < 65 years had a higher likelihood of reporting severe-to-extreme health-related financial strain (13% vs. 3%, RR: 4.3,  95% CI: 1.2–15.7), and experiencing any material hardship since the start of the 2020 pandemic (76% vs. 51%, RR: 1.5, 95% CI: 1.2–1.9), with a majority using up most or all savings (52% vs. 24%, RR: 2.2, 95% CI: 1.4–3.4).

Veterans with greater morbidity burden (i.e., Nosos risk-adjustment scores > 1) had a greater likelihood of reporting severe-to-extreme health-related financial strain since the beginning of the 2020 pandemic (14% vs. 3%, RR: 4.8, 95% CI: 1.8–13.0) than Veterans with less morbidity burden (Nosos scores ≤ 1), and having a loved one take time off work to care for them (27% vs. 14%, RR: 2.0, 95% CI: 1.1–3.7) (Figs. 3 and 4). However, they were less likely to skip or delay medical care due to cost (10% vs. 24%, RR: 0.4, 95% CI: 0.2–0.9). We observed no differences in the likelihood of financial hardship by VHA copay group, except that Veterans with VHA copays were less likely to report severe-to-extreme health-related financial strain than Veterans exempt from VHA copays (1% vs. 12%, RR: 0.10, 95% CI: 0.03–0.34) (see eTable 3, Additional File 1 for risk ratios and 95% CIs; see eTables 4–6, Additional File 1 for weighted sample characteristics by selected risk factors).

## Discussion

 Using a prospective national cohort of Veterans with a history of COVID-19 and their matched uninfected comparators, we surveyed Veterans and found that the prevalence of financial hardship was quite high, with more than two-thirds of Veteran respondents reporting at least one type of financial hardship since the beginning of the 2020 pandemic. In addition, nearly one in ten reported severe-to-extreme health-related financial strain, and two-thirds and one in five reported material and behavioral financial hardships, respectively, since the beginning of the pandemic. Veterans with a history of COVID-19 had a greater risk of reporting certain health-related financial hardships, including severe-to-extreme health-related financial strain, taking less medication due to cost, and having a loved one take time off work to care for them.

Our results suggest that financial hardship following COVID-19 infection (1) may extend well beyond the short-term period, (2) is not limited to individuals with severe COVID-19 (indicated by hospitalization), and (3) may be more common among vulnerable populations served by safety-net health systems. For example, Becker and colleagues (2023) found that approximately 15% of privately insured adults in Michigan with COVID-19 and 26% who were hospitalized with COVID-19 had some medical or non-medical debt in collections [6]. In our study and irrespective of their COVID-19 hospitalization status (6% of our sample were hospitalized at the time of infection), approximately 21% of Veterans with COVID-19 were contacted by collections. In addition, Admon and colleagues found that, within six months of a hospitalization for COVID-19, 35% of individuals in the Biology and Longitudinal Epidemiology of PETAL COVID-19 Observational Study (BLUE CORAL) had used up most or all savings, 20% were unable to pay for necessities, 16% had been contacted by collections, 9% skipped or delayed medical care due to cost, 7% took less medication due to cost, and 1.5% declared bankruptcy [5]. Up to twice as many Veterans with COVID-19 that responded to our survey reported certain of these financial hardships (e.g., 17% skipped or delayed medical care due cost; 14% took less medication due to cost; 2.5% declared bankruptcy).

Our results also suggest that certain Veteran subgroups may be at greater risk of financial hardship during public health emergencies like the COVID-19 pandemic. Specifically, Veterans of working age (< 65 years) had a greater likelihood of nearly every type of hardship that we examined compared with Veterans of retirement age (≥ 65 years). One explanation is that these younger Veterans have more exposure to employment-related financial hardship (8% of Veterans < 65 versus 75% of Veterans ≥ 65 reported being retired at the time of survey completion). In addition, younger Veterans are more likely to lack additional sources of health care coverage which may supplement their VHA benefits [37], potentially increasing their risk of health-related financial hardships [23]. However, additional research may help uncover causal mechanisms for these finding. Results were less consistent for other subgroups: Veterans with higher Nosos risk-adjustment scores were more likely to experience severe-to-extreme health-related financial strain and having a loved one take time off work to care for them, but less likely to lose a job or skip or delay medical care due of cost. Veterans with and without VHA copays had similar likelihoods of most hardships. 

These results document the sweeping impact the pandemic broadly, and COVID-19 infection specifically, has had on the financial health of US Veterans. One particularly concerning finding is the substantial number of Veterans who reported health-related financial hardships such as severe-to-extreme health-related financial strain and taking less medication than prescribed due to cost. The VHA is designed as a safety net for enrolled Veterans, with no monthly premiums, and depending on eligibility factors, low-to-no cost-sharing for covered services. Research nonetheless shows that Veterans are sensitive to VHA benefits designs such as medication copays [38, 39], and many Veterans have non-service-connected conditions which may not be covered by their VHA benefits. Further, approximately half of US Veterans have non-VHA coverage such as Medicare [40] and fewer than one-third use VHA health care each year [41]. Recent estimates indicate that nearly four in ten Medicare enrollees spends at least 20% of their income on premiums and medical expenses [42]. These health care affordability challenges may be compounded by sudden health shocks such as COVID-19. Given the serious consequences of delayed or forgone medical care and the psychological impacts of financial hardship, research is needed to understand causal mechanisms for these findings and to develop solutions for Veterans experiencing financial hardship, including following COVID-19 infection.

### Strengths and limitations

This study has several strengths. Importantly, we utilized national electronic health records and administrative data to build cohorts comprising the universe of VHA-enrolled Veterans with documented COVID-19 infections between March 2020 and April 2021 and matched comparators without a history of COVID-19. This allowed a detailed assessment of whether COVID-19 infection, versus broader economic strain, imparted financial hardship in Veterans. In addition, these data sources provided us with detailed information on the sociodemographic, clinical, and health care utilization characteristics of our sample, which can be infeasible to collect using survey methods alone. Our use of the VA COVID-19 Shared Data Resource (CSDR) is another strength of our study. The CSDR was developed through a coordinated effort of clinical, public health, and bio-surveillance experts, and includes VHA health care facility, non-VHA health care facility, and community-detected COVID-19 cases. Unlike self-reported COVID-19 infection history, which is subject to recall and reporting bias, the CSDR afforded us a high level of confidence in our ability to ascertain COVID-19 infections comprehensively and accurately at a time when health care facility-based testing was more common and home-/self-testing was more rare.

We also acknowledge limitations. This assessment of Veterans’ self-reported financial hardship would be complemented by objective measures of financial wellbeing. While our approach has the advantage of capturing outcomes typically unmeasured in secondary data and centering Veterans’ experiences, we cannot rule out recall and reporting bias. Yet, we took steps to protect from these in designing and administering the survey (e.g., using the same survey instrument for both COVID-19 and uninfected comparators, without mention of COVID-19 status). Next, our sample lacks sufficient women sex-assigned-at-birth, non-binary gender, Black/African American and other race, and Hispanic ethnicity Veterans to detect differences in hardship among and between these groups. These groups account for an increasing share of the US Veteran population and data suggest they have outsized risks for financial hardship [3, 15]. In addition, few respondents were hospitalized for COVID-19, thus we were unable to assess differences in financial hardship by infection severity. Future research should oversample these Veterans to understand their experiences and potential solutions more fully. Third, although we sought to maximize rigor using a directed acyclic graph-informed emulated target trial design [29], we cannot rule out unmeasured confounding. In addition, we did not directly measure Veterans’ baseline socioeconomic status. However, we were able to assess Veterans’ level of area deprivation suing the Area Deprivation Index (ADI), which ranks neighborhoods based on socioeconomic disadvantage [43], and found little difference between Veterans with and without COVID-19 (SMD < 0.1). Lastly, our results may not generalize beyond the VHA-engaged Veteran population.

## Conclusions

There has been a staggering burden of financial hardship among Veterans two years into the pandemic, and compared with matched uninfected comparators, a significantly greater likelihood of certain health-related hardships among Veterans with a history of COVID-19. These findings suggest post-infection sequelae go beyond some of the more commonly measured health impacts, and research and strategies are needed to better understand and devise solutions to financial hardship, including among Veterans after COVID-19 infection.

### Supplementary Information

Additional File 1. eTable 1. Unethical Target Trial Versus Emulated Target Trial Design. eTable 2. Financial Hardship Survey Questions and Response Options. eTable 3. Financial Hardship (Risk Ratio, 95% CI), by COVID-19 Status and Selected Risk Factors. eTable 4. Weighted Sample Characteristics, by Age Group. eTable 5. Weighted Sample Characteristics, by Nosos Risk-Adjustment Score Group. eTable 6. Weighted Sample Characteristics, by VHA Copay Group. eTable 7. Financial Hardship (Weighted Proportion, 95% CI), Overall and by COVID-19 Status (Excluding 8 Matched Pairs Where the Comparator Became Infected with COVID-19 Between Index and Survey Completion). eTable 8. Financial Hardship (Risk Ratio, 95% CI), by COVID-19 Status (Excluding 8 Matched Pairs Where the Comparator Became Infected with COVID-19 Between Index and Survey Completion)

## Data Availability

The United States Department of Veterans Affairs (VA) places legal restrictions on access to veteran’s health care data, which includes both identifying data and sensitive patient information. The analytic data sets used for this study are not permitted to leave the VA firewall without a Data Use Agreement. This limitation is consistent with other studies based on VA data. However, VA data are made freely available to researchers behind the VA firewall with an approved VA study protocol. For more information, please visit https://www.virec.research.va.gov or contact the VA Information Resource Center (VIReC) at VIReC@va.gov. Interested individuals may contact the corresponding author, Dr. Govier, for more information.

## Abbreviations

ADI: Area Deprivation Index
BLUE CORAL: Biology and Longitudinal Epidemiology of PETAL COVID-19 Observational Study
CDW: Corporate Data Warehouse
CI: Confidence interval
CSDR: COVID-19 Shared Data Resources
CORC: COVID-19 Observational Research Collaboratory
COVID-19: Coronavirus disease 2019
GEE: Generalized estimating equations
HSR&D: Health Services Research & Development
IRB: Institutional Review Board
RCS: Research Career Scientist
RR: Risk ratio
SARS-CoV-2: Severe acute respiratory syndrome coronavirus 2
SD: Standard deviation
SDR: Service Directed Research
SMD: Standardized mean difference
STROBE: Strengthening the reporting of observational studies in epidemiology
US: United States
VA: Veterans Affairs
VHA: Veterans Health Administration
VIReC: VA Information Resource Center
WHO: World Health Organization
