# Supplementary material for: Financial hardship after COVID-19 infection among US Veterans: a national prospective cohort study
Source: BMC Health Serv Res. 2024 Aug 19;24:943. doi: 10.1186/s12913-024-11421-1 (PMC11331641; doi:10.1186/s12913-024-11421-1)
Supplement: Supplementary file 1 — Additional File 1. eTable 1. Unethical Target Trial Versus Emulated Target Trial Design. eTable 2. Financial Hardship Survey Questions and Response Options. eTable 3. Financial Hardship (Risk Ratio, 95% CI), by COVID-19 Status and Selected Risk Factors. eTable 4. Weighted Sample Characteristics, by Age Group. eTable 5. Weighted Sample Characteristics, by Nosos Risk-Adjustment Score Group. eTable 6. Weighted Sample Characteristics, by VHA Copay Group. eTable 7. Financial Hardship (Weighted Proportion, 95% CI), Overall and by COVID-19 Status (Excluding 8 Matched Pairs Where the Comparator Became Infected with COVID-19 Between Index and Survey Completion). eTable 8. Financial Hardship (Risk Ratio, 95% CI), by COVID-19 Status (Excluding 8 Matched Pairs Where the Comparator Became Infected with COVID-19 Between Index and Survey Completion) [file 12913_2024_11421_MOESM1_ESM.docx]

**Table of Contents**

[eTable 1. Unethical Target Trial Versus Emulated Target Trial Design 2](#_Toc173323901)

[eTable 2. Financial Hardship Survey Questions and Response Options 4](#_Toc173323902)

[eTable 3. Financial Hardship (Risk Ratio, 95% CI), by COVID-19 Status and Selected Risk Factors 5](#_Toc173323903)

[eTable 4. Weighted Sample Characteristics, by Age Group 6](#_Toc173323904)

[eTable 5. Weighted Sample Characteristics, by Nosos Risk-Adjustment Score Group 8](#_Toc173323905)

[eTable 6. Weighted Sample Characteristics, by VHA Copay Group 10](#_Toc173323906)

[eTable 7. Financial Hardship (Weighted Proportion, 95% CI), Overall and by COVID-19 Status (Excluding 8 Matched Pairs Where the Comparator Became Infected with COVID-19 Between Index and Survey Completion) 12](#_Toc173323907)

[eTable8: Financial Hardship (Risk Ratio, 95% CI), by COVID-19 Status (Excluding 8 Matched Pairs Where the Comparator Became Infected with COVID-19 Between Index and Survey Completion). 13](#_Toc173323908)

# eTable 1. Unethical Target Trial Versus Emulated Target Trial Design

|  | **Unethical target trial** | **Emulated target trial** |
| --- | --- | --- |
| **Goal** | To test the effect of individual infection with SARS-CoV-2 (a.k.a. COVID-19) on financial hardship 18 months after infection | Same |
| **Setting** | VA nationwide system | Same |
| **Inclusion criteria** | Veterans aged 18 and above in care in the VHA with an assigned primary care team for at least two years on randomization date, or who had at least one VHA primary care clinic visit in that period | Same |
| **Exclusion criteria** | Previous COVID-19 Infection  Address outside of DC or 50 States | Previous documented SARS-CoV-2 Infection in National Surveillance tool or Medicare-documented COVID-19 diagnosis or related diagnostic codes (ICD-10: B97.29, U07.1, U09.9, J12.82, 179 Z86.16) listed in fee-for-service Medicare claims  Address outside of DC or 50 States  Missing or invalid key matching variables: age, height, weight, ZIP code  No suitable matches between infected patients and comparator |
| **Enrollment period** | October 2020—April, 2021 | Same |
| **“Treatment” strategies** | Inoculum of SARS-CoV-2 sufficient to guarantee COVID-19 infection | SARS-CoV-2 Infection with a confirmatory PCR test for SARS-CoV-2 in VA National Surveillance Tool |
| **Comparator** | Double-blinded inoculum of placebo | Best matched Veteran with neither documented SARS-CoV-2 Infection in National Surveillance tool nor Medicare-documented COVID-19 diagnosis through the month at which matched as a comparator |
| **Approach to balancing confounders** | 1:1 Randomization, stratified by month and center | Up to 5:1 (Comparator-to-Infected) Matching on 5 exact criteria and 37 propensity score criteria from VA data. Exact match variables included Index month (March 2020 – April 2021), sex, immunosuppressive medication use, state of residence (and Washington DC), and vaccination status (January-April 2021). Propensity score matched variables included those used in exact matching as well as nursing home residence; race; ethnicity; rurality; smoking status; CAN score; Nosos score; indicators for diagnosed CDC high-risk conditions based on ICD-19 codes: coronary heart disease, cancer (excluding non-metastatic skin cancers), chronic kidney disease, congestive heart failure, pulmonary-associated conditions (including asthma, COPD, interstitial lung disease, and cystic fibrosis), dementia, diabetes, hypertension, liver disease, sickle cell/thalassemia, solid organ or blood stem cell transplant, stroke/cerebrovascular disorders, substance use disorder, anxiety disorder, bipolar disorder, major depression, PTSD, and schizophrenia; age; body mass index; comorbidity score via Gagne index; number VHA inpatient admissions in two years prior to index; number VHA primary care visits in two years prior to index; number VHA specialty care visits in two years prior to index; number VHA mental health visits in two years prior to index; and distance to nearest VA medical center. |
| **Primary outcome** | Self-reported financial hardship | Same |
| **Follow-up period** | 18 months from inoculation | 18 months from the earliest date of a documented positive test for those with COVID-19 infection; comparators began surveillance for outcomes from the same date (“index date”, the emulated equivalent of “randomization and inoculation date”) as that of their individually matched infected patient and were followed for 18 months |
| **Causal contrast** | Primary Analysis: comparison of outcomes between survivors of COVID-19 and those without contemporaneous infection | Primary Analysis: same |

eTable 1 Footnote: Abbreviations: VHA: Veterans Health Administration; SARS-CoV-2: severe acute respiratory syndrome coronavirus version 2; PCR: polymerase chain reaction; COVID-19: Coronavirus disease 2019; ICD-10: International classification of diseases tenth revision clinical modification; CAN: Care Assessment of Need Score

# eTable 2. Financial Hardship Survey Questions and Response Options

| **Survey question** | **Response options** |
| --- | --- |
| Material financial hardships | |
| Since the beginning of the 2020 pandemic have you used up all or most of your savings? | Yes, No |
| Since the beginning of the 2020 pandemic were you unable to pay for necessities like food, heat, or housing? | Yes, No |
| Since the beginning of the 2020 pandemic have you been contacted by a collection agency? | Yes, No |
| Since the beginning of the 2020 pandemic have you declared bankruptcy? | Yes, No |
| Since the beginning of the 2020 pandemic have you lost a job? | Yes, No |
| Since the beginning of the 2020 pandemic have you had to change the kind of work you can do? | Yes, No |
| Since the beginning of the 2020 pandemic have you had a loved one take time off work to care for you? | Yes, No |
| Behavioral financial hardships | |
| Since the beginning of the 2020 pandemic have you skipped or delayed getting medical care you thought you needed because of the cost? | Yes, No |
| Since the beginning of the 2020 pandemic have you taken less medication than was prescribed to you because of the cost? | Yes, No |
| World Health Organization assessment instrument of health-related financial strain | |
| Since the beginning of the 2020 pandemic how much has your health been a drain on the financial resources of you or your family? | None, Mild, Moderate, Severe, Extreme |

# eTable 3. Financial Hardship (Risk Ratio, 95% CI), by COVID-19 Status and Selected Risk Factors

| **Financial Hardship Outcome** | **History of COVID-19**  **(vs. no history of COVID-19)** | **Aged <65**  **(vs. aged ≥65)** | **Nosos score >1**  **(vs. Nosos Score ≤1)** | **Some/full VHA copay**  **(vs. no VHA copay)** |
| --- | --- | --- | --- | --- |
| Any financial hardship | 1.0 (0.8 – 1.3) | 1.4 (1.2 – 1.8) | 1.1 (0.9 – 1.3) | 0.9 (0.7 – 1.2) |
| Severe-to-extreme health-related financial strain | 4.0 (1.4 – 11.2) | 4.3 (1.2 – 15.7) | 4.8 (1.8 – 13.0) | 0.1 (0.0 – 0.3) |
| Any behavioral financial hardship | 1.0 (0.5 – 1.9) | 2.1 (1.1 – 4.2) | 0.5 (0.2 – 0.9) | 1.0 (0.6 – 2.0) |
| Skipped or delayed medical care | 0.9 (0.5 – 1.8) | 3.5 (1.6 – 7.6) | 0.4 (0.2 – 0.9) | 0.9 (0.4 – 1.7) |
| Took less medication | 2.9 (1.0 – 8.6) | 1.0 (0.4 – 2.7) | 0.7 (0.2 – 1.8) | 0.8 (0.3 – 2.3) |
| Any material financial hardship | 1.0 (0.8 – 1.3) | 1.5 (1.2 – 1.9) | 1.1 (0.9 – 1.4) | 1.0 (0.8 – 1.2) |
| Used up most or all savings | 1.2 (0.9 – 1.7) | 2.2 (1.4 – 3.4) | 1.4 (0.9 – 2.0) | 1.1 (0.8 – 1.6) |
| Changed kind of work | 1.3 (0.8 – 2.1) | 1.8 (1.0 – 3.2) | 1.1 (0.7 – 1.7) | 0.7 (0.4 – 1.3) |
| Contacted by collections | 0.9 (0.5 – 1.5) | 3.4 (1.7 – 6.6) | 1.0 (0.6 – 1.7) | 0.9 (0.5 – 1.7) |
| Loved one took time off work | 1.9 (1.1 – 3.6) | 1.6 (0.9 – 3.1) | 2.0 (1.1 – 3.7) | 0.5 (0.2 – 1.0) |
| Unable to pay for necessities | 0.9 (0.6 – 1.5) | 1.9 (1.1 – 3.4) | 1.7 (0.9 – 3.1) | 0.9 (0.5 – 1.8) |
| Lost a job | 1.8 (0.7 – 4.3) | 5.5 (1.6 – 18.2) | 0.4 (0.2 – 1.0) | 1.1 (0.5 – 2.5) |
| Declared bankruptcy | 1.2 (0.2 – 7.6) | 1.0 (0.2 – 6.2) | 2.6 (0.5 – 13.5) | 2.2 (0.4 – 11.3) |

eTable 3 Footnote: Abbreviations: CI: 95% Confidence interval; VHA: Veterans Health Administration

# eTable 4. Weighted Sample Characteristics, by Age Group

| **Baseline characteristic (weighted %)** | **Aged <65  N = 224** | **Aged ≥65  N = 164** | **SMD** |
| --- | --- | --- | --- |
| **Used in matching Veterans with history of COVID-19 to uninfected comparators** | | | |
| COVID-19 | 53.8 | 46.1 | 0.154 |
| Sex |  |  | 0.474 |
| Female | 15.6 | 2.4 |  |
| Male | 84.4 | 97.6 |  |
| Unknown | 0.1 | 0.1 |  |
| Age, years, mean (SD) | 49.61 (9.3) | 72.55 (5.7) | -2.990 |
| Race |  |  | 0.275 |
| Black or African American | 31.9 | 20.2 |  |
| White | 62.1 | 74.3 |  |
| Other | 6.0 | 5.5 |  |
| Ethnicity |  |  | 0.076 |
| Hispanic | 7.4 | 5.7 |  |
| Not Hispanic | 90.8 | 92.0 |  |
| Unknown | 1.8 | 2.3 |  |
| Smoking status |  |  | 0.557 |
| Current | 7.1 | 14.2 |  |
| Former | 35.3 | 54.3 |  |
| Never | 50.0 | 28.9 |  |
| Unknown | 7.5 | 2.6 |  |
| Vaccinated for COVID-19 January - April 2021 |  |  | 0.123 |
| Unvaccinated | 2.0 | 2.2 |  |
| Vaccinated | 29.5 | 24.0 |  |
| Vaccine unavailable at index date | 68.6 | 73.8 |  |
| Urban residence | 73.4 | 61.3 | 0.260 |
| Nosos risk-adjustment score |  |  | 0.161 |
| ≤1 | 56.9 | 50.0 |  |
| >1 | 42.1 | 49.5 |  |
| Unknown | 1.0 | 0.4 |  |
| Immunosuppressed | 10.3 | 11.0 | -0.024 |
| No. VHA inpatient admissions, mean (SD) | 0.3 (1.1) | 0.4 (1.1) | -0.102 |
| No. VHA primary care visits, mean (SD) | 8.6 (9.3) | 12.9 (12.3) | -0.388 |
| No. VHA specialty care visits, mean (SD) | 13.4 (13.6) | 20.0 (19.7) | -0.389 |
| No. VHA mental health visits, mean (SD) | 8.7 (17.6) | 3.8 (14.6) | 0.303 |
| BMI, mean (SD) | 32.9 (6.6) | 30.5 (6.4) | 0.374 |
| Gagne index, mean (SD) | 0.6 (1.4) | 1.7 (2.3) | -0.580 |
| CAN score, mean (SD) | 46.1 (28.9) | 68.4 (22.2) | -0.869 |
| Distance to nearest VAMC, miles, mean (SD) | 33.7 (35.4) | 36.9 (35.4) | -0.091 |
|  |  |  |  |
| **Not used in matching Veterans with history of COVID-19 to uninfected comparators** | | | |
| No. CDC high-risk conditions, mean (SD) | 1.4 (1.2) | 2.8 (1.9) | -0.892 |
| No. CDC high-risk mental health conditions, mean (SD) | 1.1 (1.1) | 0.5 (0.8) | 0.723 |
| Hospitalized at index date (+/- 7 days) | 3.1 | 8.8 | -0.243 |
| VHA copay group |  |  | 0.673 |
| Full copay | 11.7 | 23.9 |  |
| Some copay | 10.4 | 28.8 |  |
| No copay | 78.0 | 47.3 |  |
| Area Deprivation Index, mean (SD) | 53.1 (22.5) | 59.4 (25.9) | -0.260 |
| Area Deprivation Index Median |  |  | 0.120 |
| Lower Median (1-50) | 44.4 | 47.3 |  |
| Upper Median (51-100) | 52.7 | 58.6 |  |
| **Characteristic at time of survey completion (weighted %)** | **Aged <65  N = 224** | **Aged ≥65  N = 164** | **SMD** |
| Employment status |  |  | 1.900 |
| Currently working | 61.5 | 12.0 |  |
| Unemployed and looking for work | 6.6 | 4.3 |  |
| Disabled | 23.9 | 8.9 |  |
| Retired/Other | 8.0 | 74.8 |  |

eTable 4 Footnote: Other baseline variables used in matching Veterans with history of COVID-19 to uninfected comparators but not reported to preserve table brevity include index month, state of residence, and indicators for nursing home residence, coronary heart disease, cancer (excluding non-metastatic skin cancers), chronic kidney disease, congestive heart failure, pulmonary-associated conditions (including asthma, COPD, interstitial lung disease, and cystic fibrosis), dementia, diabetes, hypertension, liver disease, sickle cell/thalassemia, solid organ or blood stem cell transplant, stroke/cerebrovascular disorders, substance use disorder, anxiety disorder, bipolar disorder, major depression, PTSD, and schizophrenia. Abbreviations: SMD: Standardized mean difference; BMI: Body Mass Index; CAN: Care Assessment Need; CDC: Centers for Disease Control and Prevention; VHA: Veterans Health Administration; VAMC: VA Medical Center; SD: Standard Deviation.

# eTable 5. Weighted Sample Characteristics, by Nosos Risk-Adjustment Score Group

| **Baseline characteristic (weighted %)** | **Nosos score ≤ 1**  **N = 204** | **Nosos score >1**  **N = 174** | **SMD** |
| --- | --- | --- | --- |
| **Used in matching Veterans with history of COVID-19 to uninfected comparators** | | | |
| COVID-19 | 50.5 | 49.7 | 0.017 |
| Sex |  |  |  |
| Female | 8.4 | 10.0 |  |
| Male | 91.5 | 90.0 |  |
| Unknown | 0.1 | 0.0 |  |
| Age, years, mean (SD) | 59.2 (15.3) | 62.9 (11.5) | -0.272 |
| Race |  |  | 0.042 |
| Black or African American | 26.7 | 25.8 |  |
| White | 67.9 | 67.9 |  |
| Other | 5.4 | 6.3 |  |
| Ethnicity |  |  | 0.191 |
| Hispanic | 7.5 | 5.2 |  |
| Not Hispanic | 89.4 | 93.9 |  |
| Unknown | 3.1 | 0.8 |  |
| Smoking status |  |  | 0.425 |
| Current | 12.5 | 8.5 |  |
| Former | 36.9 | 53.6 |  |
| Never | 42.5 | 36.2 |  |
| Unknown | 8.1 | 1.7 |  |
| Vaccinated for COVID-19 January - April 2021 |  |  | 0.228 |
| Unvaccinated | 2.8 | 1.3 |  |
| Vaccinated | 30.1 | 21.7 |  |
| Vaccine unavailable at index date | 67.1 | 77.0 |  |
| Urban residence | 68.8 | 66.0 | 0.059 |
| Nosos risk-adjustment score |  |  |  |
| ≤1 | 100.0 | 0.0 |  |
| >1 | 0.0 | 100.0 |  |
| Immunosuppressed | 1.8 | 21.1 | -0.637 |
| No. VHA inpatient admissions, mean (SD) | 0.1 (0.7) | 0.7 (1.4) | -0.534 |
| No. VHA primary care visits, mean (SD) | 6.1 (7.8) | 16.3 (11.9) | -1.030 |
| No. VHA specialty care visits, mean (SD) | 8.1 (8.5) | 26.9 (19.1) | -1.280 |
| No. VHA mental health visits, mean (SD) | 2.7 (7.0) | 10.5 (22.3) | -0.477 |
| BMI, mean (SD) | 31.1 (6.3) | 32.4 (6.9) | -0.198 |
| Gagne index, mean (SD) | 0.6 (1.4) | 1.9 (2.3) | -0.643 |
| CAN score, mean (SD) | 40.6 (24.9) | 75.9 (17.9) | -1.630 |
| Distance to nearest VAMC, miles, mean (SD) | 40.3 (39.6) | 29.2 (28.6) | 0.324 |
| **Not used in matching Veterans with history of COVID-19 to uninfected comparators** | | | |
| No. CDC high-risk conditions, mean (SD) | 1.6 (1.5) | 2.8 (1.7) | -0.743 |
| No. CDC high-risk mental health conditions, mean (SD) | 0.7 (0.9) | 1.0 (1.1) | -0.307 |
| Hospitalized at index date (+/- 7 days) | 3.0 | 9.3 | -0.265 |
| VHA copay group |  |  | 0.447 |
| Full copay | 24.9 | 9.4 |  |
| Some copay | 20.1 | 18.3 |  |
| No copay | 54.9 | 72.3 |  |
| Area Deprivation Index, mean (SD) | 51.2 (22.5) | 62.1 (25.2) | -0.456 |
| Area Deprivation Index Median |  |  | 0.308 |
| Lower Median (1-50) | 51.3 | 36.2 |  |
| Upper Median (51-100) | 48.7 | 63.8 |  |
| **Characteristic at time of survey completion (weighted %)** | **Nosos score ≤ 1**  **N = 204** | **Nosos score >1**  **N = 174** | **SMD** |
| Employment status at time of survey completion |  |  | 0.789 |
| Currently Working | 48.1 | 24.0 |  |
| Unemployed and looking for work | 7.7 | 2.9 |  |
| Disabled | 5.6 | 29.7 |  |
| Retired/Other | 38.6 | 43.4 |  |

eTable 5 Footnote: Records without a Nosos score (N=10) were excluded. Other baseline variables used in matching Veterans with history of COVID-19 to uninfected comparators but not reported to preserve table brevity include index month, state of residence, and indicators for nursing home residence, coronary heart disease, cancer (excluding non-metastatic skin cancers), chronic kidney disease, congestive heart failure, pulmonary-associated conditions (including asthma, COPD, interstitial lung disease, and cystic fibrosis), dementia, diabetes, hypertension, liver disease, sickle cell/thalassemia, solid organ or blood stem cell transplant, stroke/cerebrovascular disorders, substance use disorder, anxiety disorder, bipolar disorder, major depression, PTSD, and schizophrenia. Abbreviations: SMD: Standardized mean difference; BMI: Body Mass Index; CAN: Care Assessment Need; CDC: Centers for Disease Control and Prevention; VHA: Veterans Health Administration; VAMC: VA Medical Center; SD: Standard Deviation.

# eTable 6. Weighted Sample Characteristics, by VHA Copay Group

| **Baseline characteristic (weighted %)** | **No VHA copay N = 262** | **Some/Full VHA copay N = 126** | **SMD** |
| --- | --- | --- | --- |
| **Used in matching Veterans with history of COVID-19 to uninfected comparators** | | | |
| COVID-19 | 48.9 | 51.8 | -0.058 |
| Sex |  |  | 0.259 |
| Female | 11.7 | 4.7 |  |
| Male | 88.2 | 95.2 |  |
| Unknown | 0.0 | 0.1 |  |
| Age, years, mean (SD) | 57.5 (13.4) | 66.6 (12.6) | -0.706 |
| Race |  |  | 0.152 |
| Black or African American | 24.8 | 28.4 |  |
| White | 70.4 | 64.1 |  |
| Other | 4.8 | 7.5 |  |
| Ethnicity |  |  | 0.124 |
| Hispanic | 7.4 | 5.2 |  |
| Not Hispanic | 91.1 | 92.0 |  |
| Unknown | 1.6 | 2.8 |  |
| Smoking status |  |  | 0.184 |
| Current | 9.7 | 12.0 |  |
| Former | 43.2 | 47.2 |  |
| Never | 42.7 | 34.4 |  |
| Unknown | 4.4 | 6.4 |  |
| Vaccinated for COVID-19 January - April 2021 |  |  | 0.303 |
| Unvaccinated | 3.2 | 0.2 |  |
| Vaccinated | 29.4 | 22.3 |  |
| Vaccine unavailable at index date | 67.4 | 77.5 |  |
| Urban residence | 65.7 | 70.4 | 0.101 |
| Nosos risk-adjustment score |  |  | 0.379 |
| ≤1 | 46.7 | 65.1 |  |
| >1 | 52.6 | 34.2 |  |
| Unknown | 0.7 | 0.8 |  |
| Immunosuppressed | 11.9 | 8.4 | 0.115 |
| No. VHA inpatient admissions, mean (SD) | 0.4 (1.2) | 0.4 (1.0) | 0.031 |
| No. VHA primary care visits, mean (SD) | 10.7 (11.3) | 10.7 (10.7) | 0.005 |
| No. VHA specialty care visits, mean (SD) | 17.2 (17.7) | 15.7 (16.1) | 0.088 |
| No. VHA mental health visits, mean (SD) | 8.3 (18.7) | 2.7 (10.5) | 0.372 |
| BMI, mean (SD) | 32.5 (6.4) | 30.3 (6.7) | 0.334 |
| Gagne index, mean (SD) | 1.0 (2.0) | 1.4 (2.0) | -0.198 |
| CAN score, mean (SD) | 56.0 (28.1) | 58.7 (28.2) | -0.097 |
| Distance to nearest VAMC, miles, mean (SD) | 36.4 (35.6) | 33.4 (35.1) | 0.085 |
| **Not used in matching Veterans with history of COVID-19 to uninfected comparators** | | | |
| No. CDC high-risk conditions, mean (SD) | 1.9 (1.6) | 2.5 (1.9) | -0.319 |
| No. CDC high-risk mental health conditions, mean (SD) | 1.0 (1.1) | 0.4 (0.8) | 0.635 |
| Hospitalized at index date (+/- 7 days) | 6.3 | 5.1 | 0.051 |
| VHA copay group |  |  |  |
| Full copay | 0.0 | 47.6 |  |
| Some copay | 0.0 | 52.4 |  |
| No copay | 100.0 | 0.0 |  |
| Area Deprivation Index, mean (SD) | 56.7 (23.8) | 55.2 (25.5) | 0.061 |
| Area Deprivation Index Median |  |  | 0.044 |
| Lower Median (1-50) | 43.6 | 45.8 |  |
| Upper Median (51-100) | 56.4 | 54.2 |  |
| **Characteristic at time of survey completion (weighted %)** | **No VHA copay N = 262** | **Some/Full VHA copay N = 126** | **SMD** |
| Employment status |  |  | 0.583 |
| Currently working | 37.7 | 36.4 |  |
| Unemployed and looking for work | 7.0 | 2.9 |  |
| Disabled | 22.5 | 6.5 |  |
| Retired/Other | 32.9 | 54.1 |  |

eTable 6 Footnote: Other baseline variables used in matching Veterans with a history of COVID-19 to uninfected comparators but not reported to preserve table brevity include index month, state of residence, and indicators for nursing home residence, coronary heart disease, cancer (excluding non-metastatic skin cancers), chronic kidney disease, congestive heart failure, pulmonary-associated conditions (including asthma, COPD, interstitial lung disease, and cystic fibrosis), dementia, diabetes, hypertension, liver disease, sickle cell/thalassemia, solid organ or blood stem cell transplant, stroke/cerebrovascular disorders, substance use disorder, anxiety disorder, bipolar disorder, major depression, PTSD, and schizophrenia. Abbreviations: SMD: Standardized mean difference; BMI: Body Mass Index; CAN: Care Assessment Need; CDC: Centers for Disease Control and Prevention; VHA: Veterans Health Administration; VAMC: VA Medical Center; SD: Standard Deviation.

# eTable 7. Financial Hardship (Weighted Proportion, 95% CI), Overall and by COVID-19 Status (Excluding 8 Matched Pairs Where the Comparator Became Infected with COVID-19 Between Index and Survey Completion)

| **Financial Hardship Outcome** | **Overall** | **History of COVID-19** | **Matched comparator** | **SMD** |
| --- | --- | --- | --- | --- |
| Any financial hardship | 67.0 (59.3 – 73.8) | 67.4 (56.1 – 76.9) | 66.6 (55.9 – 75.8) | 0.016 |
| Health-related financial strain |  |  |  | 0.270 |
| None | 42.0 (34.6 – 49.9) | 37.4 (27.6 – 48.4) | 46.7 (35.9 – 57.7) |  |
| Mild | 24.0 (18.0 – 31.1) | 25.5 (16.9 – 36.5) | 22.4 (15.0 – 32.2) |  |
| Moderate | 26.6 (20.1 – 34.4) | 25.6 (16.8 – 36.9) | 27.7 (18.7 – 39.0) |  |
| Severe | 2.5 (1.4 – 4.4) | 3.6 (1.7 – 7.4) | 1.3 (0.5 – 3.4) |  |
| Extreme | 4.9 (2.5 – 9.5) | 7.9 (3.6 – 16.5) | 1.9 (0.5 – 7.1) |  |
| Severe-to-Extreme | 7.4 (4.5 – 11.9) | 11.5 (6.4 – 19.8) | 3.2 (1.3 – 7.7) | 0.321 |
| Any behavioral financial hardship | 20.9 (15.2 – 27.9) | 21.6 (13.9 – 31.9) | 20.2 (12.6 – 30.6) | 0.034 |
| Skipped or delayed medical care | 18.1 (12.9 – 24.8) | 18.1 (11.2 – 27.8) | 18.2 (11.2 – 28.2) | -0.004 |
| Took less medication | 9.6 (5.91 – 15.3) | 14.4 (8.12 – 24.2) | 4.9 (1.97 – 11.5) | 0.326 |
| Any material financial hardship | 64.5 (56.8 – 71.5) | 64.5 (53.2 – 74.3) | 64.6 (53.8 – 74.0) | -0.002 |
| Used up most or all savings | 38.9 (31.5 – 46.8) | 41.2 (30.9 – 52.4) | 36.5 (26.4 – 48.0) | 0.096 |
| Changed kind of work | 24.9 (18.8 – 32.2) | 27.7 (19.0 – 38.5) | 22.2 (14.4 – 32.6) | 0.128 |
| Contacted by collections | 22.7 (16.9 – 29.9) | 19.9 (12.8 – 29.5) | 25.6 (17.1 – 36.6) | -0.138 |
| Loved one took time off work | 19.8 (14.4 – 26.6) | 25.2 (16.9 – 35.8) | 14.4 (8.40 – 23.6) | 0.274 |
| Unable to pay for necessities | 19.9 (14.1 – 27.2) | 19.1 (11.6 – 29.7) | 20.6 (12.7 – 31.8) | -0.039 |
| Lost a job | 13.0 (8.5 – 19.4) | 17.4 (10.3 – 27.9) | 8.6 (4.1 – 17.3) | 0.265 |
| Declared bankruptcy | 2.3 (1.0 – 5.5) | 2.5 (1.02 – 6.1) | 2.1 (0.4 – 10.1) | 0.027 |

eTable 7 Footnote: Abbreviations: CI: 95% Confidence interval; SMD: Standard mean difference

# eTable8: Financial Hardship (Risk Ratio, 95% CI), by COVID-19 Status (Excluding 8 Matched Pairs Where the Comparator Became Infected with COVID-19 Between Index and Survey Completion).

| **Financial Hardship Outcome** | **History of COVID-19 (vs. no history of COVID-19)** |
| --- | --- |
| Any financial hardship | 1.0 (0.8, 1.2) |
| Severe-to-extreme health-related financial strain | 3.6 (1.3, 10.1) |
| Any behavioral financial hardship | 1.1 (0.6, 2.1) |
| Skipped or delayed medical care | 1.0 (0.5, 1.9) |
| Took less medication | 2.9 (1, 8.6) |
| Any material financial hardship | 1.0 (0.8, 1.2) |
| Used up most or all savings | 1.1 (0.8, 1.6) |
| Changed kind of work | 1.2 (0.8, 2) |
| Contacted by collections | 0.8 (0.4, 1.3) |
| Loved one took time off work | 1.8 (0.9, 3.2) |
| Unable to pay for necessities | 0.9 (0.6, 1.5) |
| Lost a job | 2.0 (0.8, 5.2) |
| Declared bankruptcy | 1.2 (0.2, 7.6) |

eTable 8 Footnote: Abbreviations: CI: 95% Confidence interval; VHA: Veterans Health Administration
